# Use of ultrasonication to increase germination rates of Arabidopsis seeds

**DOI:** 10.1186/s13007-017-0182-6

**Published:** 2017-04-26

**Authors:** Ignacio López-Ribera, Carlos M. Vicient

**Affiliations:** Centre for Research in Agricultural Genomics (CRAG) CSIC-IRTA-UAB-UB, Campus UAB Bellaterra, 08193 Barcelona, Spain

**Keywords:** Ultrasound, Arabidopsis, Germination, Seed, Ageing

## Abstract

**Background:**

*Arabidopsis thaliana* is widely used as model organism in plant biology. Although not of agronomic significance, it offers important advantages for basic research in genetics and molecular biology including the availability of a large number of mutants and genetically modified lines. However, Arabidopsis seed longevity is limited and seeds stored for more than 10 years usually show a very low capacity for germination.

**Results:**

The influence of ultrasonic stimulation was investigated on the germination of *A. thaliana* L. seeds. All experiments have been performed using a frequency of 45 kHz at constant temperature (24 °C). No germination rate differences were observed when using freshly collected seeds. However, using artificially deteriorated seeds, our results show that short ultrasonic stimulation (<1 min) significantly increased germination. Ultrasonic stimulation application of 30 s is the optimal treatment. A significant increase in the germination rate was also verified in naturally aged seeds after ultrasonic stimulation. Scanning electron microscopy observations showed an increase in the presence of pores in the seed coat after sonication that may be the cause, at least in part, of the increase in germination. The ultrasound treated seeds developed normally to mature fertile plants.

**Conclusions:**

Ultrasound technology can be used to enhance the germination process of old Arabidopsis seeds without negatively affecting seedling development. This effect seems to be, at least in part, due to the opening of pores in the seed coat. The use of ultrasonic stimulation in Arabidopsis seeds may contribute to the recovering of long time stored lines.

## Background

*Arabidopsis thaliana* L. is an annual weed from the *Brassicaceae* family that lives in mild to cold climates and is widely distributed in Europe, North America and Asia. *A. thaliana* L. is universally recognized as a model for plant molecular biology and genetic studies. Although it is a non-commercial plant, it is favored among basic scientists because its short lifetime cycle, it is easy and inexpensive to grow, produces many seeds and contains a comparatively small genome. This allows large genetic experiments often involving thousands of plants [1]. Unfortunately, Arabidopsis as a model plant has also some disadvantages, being probably one of the worst the difficulties in the long-term seed conservation without loss of viability [2]. When the *A. thaliana* seeds are stored for more than 10 years usually they retain low capacity for germination.

Many invigoration treatments of seeds, referred to as seed priming, have been used to increase and/or accelerate germination [3], as, for example, the addition of chemicals, plant hormones or by controlled hydration. The addition of gibberellic acid is probably the most effective method, but is time consuming and relatively expensive. Other chemical methods may add undesirable residues to the culture. Germination may also be stimulated by physical methods as, for example, heat treatments, ionizing radiation [4] or vacuum [5].

Sound is a vibration that propagates as a mechanical wave of pressure through a medium such as air or water. Sound that is perceptible by humans has frequencies from 20 to 20,000 Hz. Ultrasounds are acoustic waves at frequencies higher than 20 kHz. Ultrasounds are often used in the agro-industry in order to enhance processes such as drying, extraction, emulsification and defoaming [6]. Ultrasound treatments have been reported to stimulate germination in different types of plants, such as *Calanthe* hybrids, bean [7], corn [8], barley [5], fern spores [9], alfalfa and broccoli [10], chickpea [11, 12], sorghum [13], navy beans [14], wheat, watermelon and pepper [15, 16], but not in Arabidopsis. This research investigates the effects of ultrasound treatments on germination of Arabidopsis seeds.

## Results

**Fig. 1:**
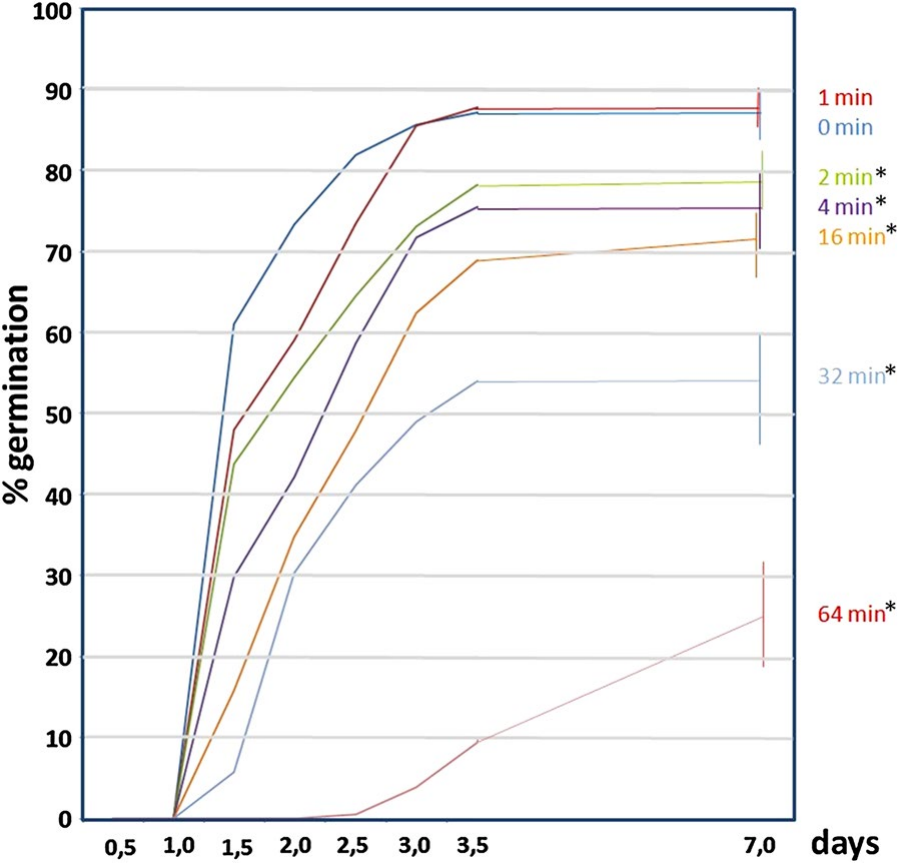
Effect of ultrasounds on the germination percentage of seeds of *A. thaliana*. 30 seeds were subjected to 45 kHz sonication at 24 °C during the indicated periods of time. *Each point* shows a mean of ten independent samples. *Vertical bars* indicate standard error. For clarity, SD is only shown for the 7 days data. *Asterisks* indicate significant differences respect to the control (untreated seeds) according to the *t* test (p < 0.05)

To determine the effect of ultrasounds on the germinability of Arabidopsis seeds, recently collected seeds were subjected to different times of sonication, and germination (radicle protrusion) was assessed at different times (1, 1.5, 2, 2.5, 3, 3.5, 7 days) (Fig. 1). Short sonication (1 min) has no significant effect on germination compared to the control, but 2 min or longer sonication treatments led to a decline of germination. Only 2 min of ultrasounds reduced germination in about 10%. Surprisingly, about 25% of the seeds treated during 1 h were able to germinate after 7 days, however, none of these seedlings were able to develop a mature plant. On the contrary, all the seeds treated during one minute developed normal and fertile plants.

**Fig. 2:**
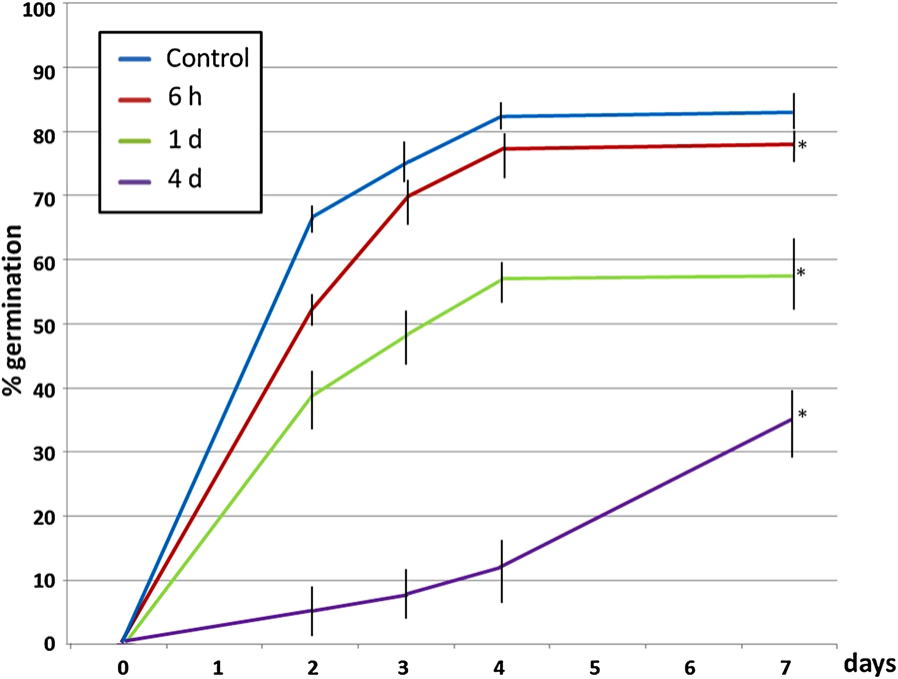
Effect of ageing treatment (40 °C and 100% relative humidity) on the germination percentage of seeds of *A. thaliana*. 30 seeds were subjected to the ageing treatment during the indicated periods of time. *Each point* shows a mean of ten independent samples. *Vertical bars* indicate standard error. *Asterisks* indicate significant differences respect to the control (untreated seeds) according to the *t* test (p < 0.05)

Next, we tested if ultrasounds increases the germination rate of aged seeds. First, we used artificially aged Arabidopsis seeds that were subjected to aging treatment for different periods of time (6 h–4 days). The time courses of germination varied depending on the length of the ageing treatment (Fig. 2). The 6 h treatment reduced germination at 7 days only about 5% respect to the control non-aged seeds. The 1 and 4 days ageing treatments produced a much more dramatic effect on seed germination with reductions of approximately 30 and 50%, respectively, after 7 days (Fig. 2). The seeds subjected to 4 days of artificially ageing treatment were selected for the subsequent assays.

**Fig. 3:**
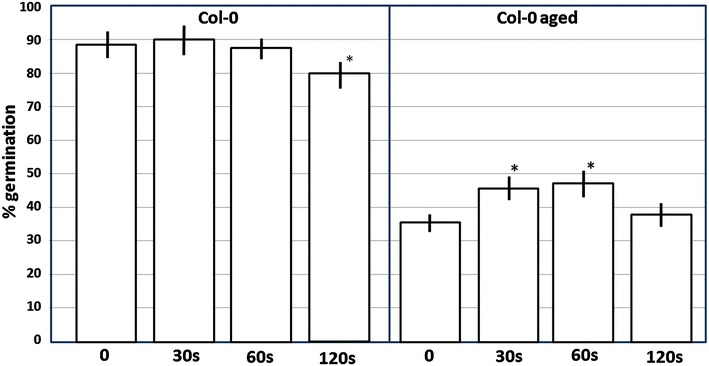
Effect of ultrasounds on the germination percentage of seeds of *A. thaliana* (Col-0) and on the same seeds that have been artificially aged. Artificial ageing treatment consisted in 4 days at 40 °C and 100% relative humidity (Col-0 aged). 30 seeds were subjected to 45 kHz sonication at 24 °C during the indicated periods of time. *Each point* shows a mean of ten independent samples. *Vertical bars* indicate standard error. *Asterisks* indicate significant differences respect to the control (the corresponding ultrasound untreated seeds) according to the *t* test (p < 0.05)

We performed ultrasounds treatments on artificially aged and non-aged seeds using three different sonication times: 30 s, 1 and 2 min. As we previously noted, no significant differences were observed in recently collected seeds except for the 2 min treated seeds in which an approximately 10% reduction in germination was observed (Fig. 3). Different results were observed for the artificially aged seeds. First, the seeds subjected to the artificial aged treatment showed a strong reduction in their germinability compared to the non-treated seeds. Second, short ultrasound treatments (30 and 60 s) produced significant (p < 0.05) increases of approximately 10% in the germinability compared to the non-ultrasound treated seeds (Fig. 3). Aged seeds treated for two minutes showed a similar germination percentage as aged seed controls.

**Fig. 4:**
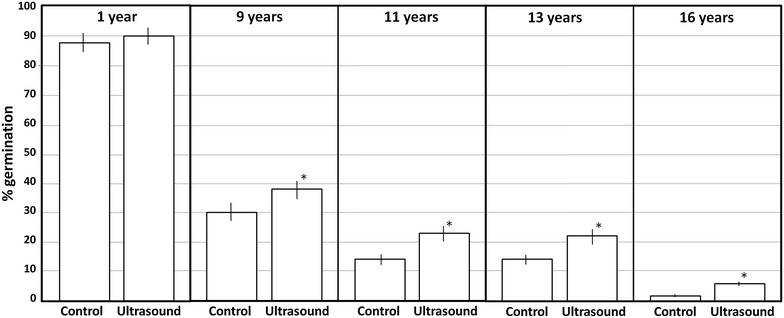
Effect of ultrasounds on the germination percentage of seeds of *A. thaliana* (Col-0) collected at different years before testing. 30 seeds were subjected to 45 kHz sonication at 24 °C for 30 s. *Each point* shows a mean of 30 independent samples. *Vertical bars* indicate standard error. *Asterisks* indicate significant differences respect to the control (the corresponding ultrasound untreated seeds) according to the *t* test (p < 0.05)

Next, we tested if ultrasound treatment also induces an increase in the germination rate of “naturally” aged Arabidopsis seeds. Arabidopsis seeds collected 1, 9, 11, 13 and 16 years ago and keep at room temperature in 1.5 ml plastic tubes were tested. In this case we chose a treatment time of 30 s because although 60 s gave a slightly better result in artificially aged seeds the difference was minimal and not significant, and in that way we tried to minimize any possible side effect that the ultrasound treatment could produce. As expected, germination rates decreased with age (Fig. 4). Except for the 1-year old seeds, in all the other cases the sonicated seeds showed a significant increase in the germination rate, being the differences higher in the 11 and 13 years old seeds.

**Fig. 5:**
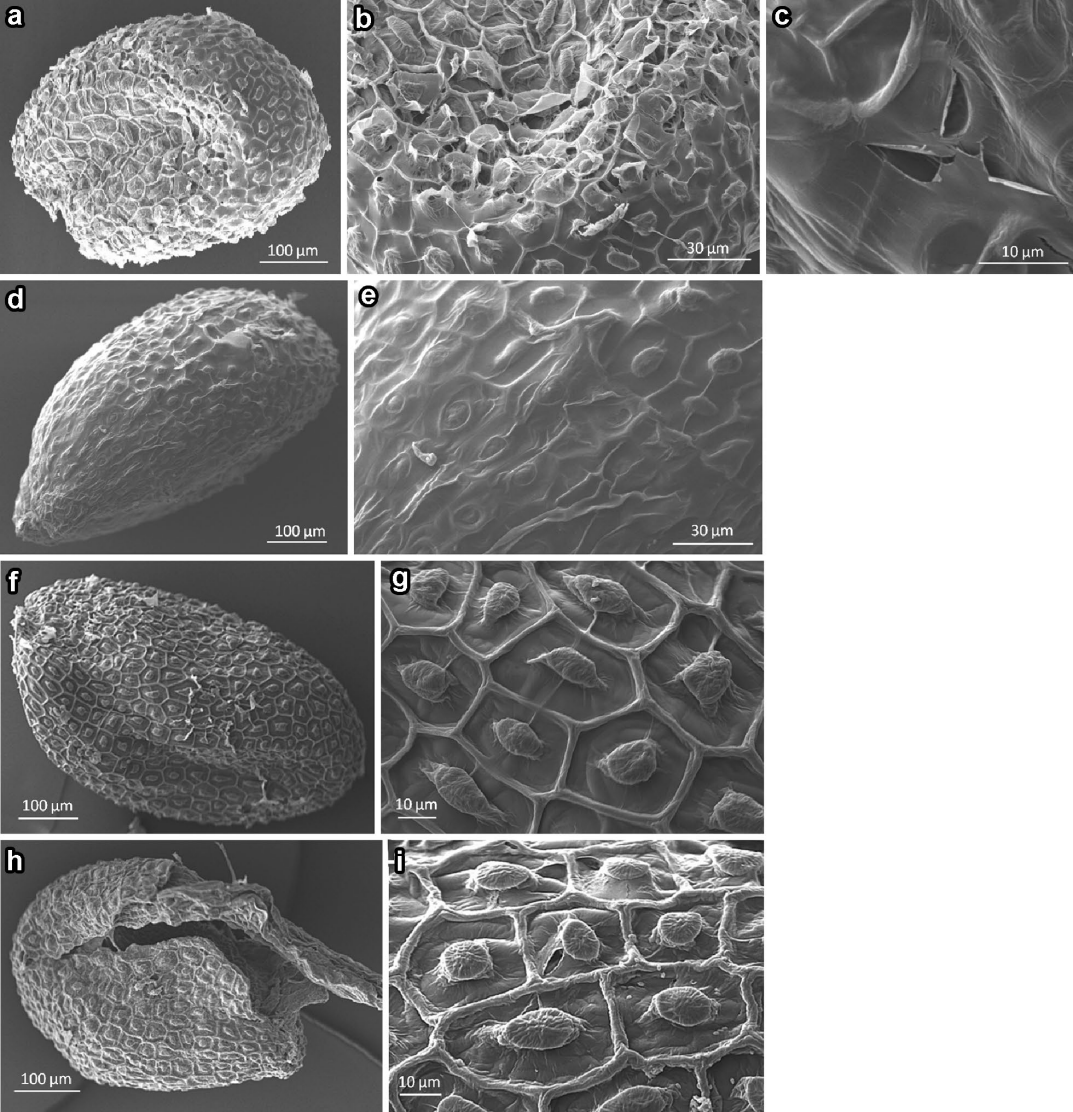
Scanning electron microscopic view of Arabidopsis Col-0 seeds. **a**–**c** Seed subjected to 45 kHz sonication at 24 °C for 30 s. **d**, **e** dry seed, **f**, **g** seed placed in water for 4 days at 4 °C so they are embedded but they do not germinate, **h**, **i**, germinated seeds

We closely examined the control and ultrasound treated Arabidopsis seeds using scanning electron microscopy (SEM) (Fig. 5). The surface of the ultrasound treated seeds presented many small pores (Fig. 5a–c). These pores are not present in the surface of the dry seeds (Fig. 5d, e) or embedded non-germinating untreated seeds (Fig. 5f, g), and are only present in small number in the untreated germinating seeds (Fig. 5h, i).

## Discussion

Seed ageing decreases the quality of seeds and results in agricultural and economic losses, and it is also a serious problem in the research laboratories. *A. thaliana* is widely used in plant basic research. It was the first plant whose complete genome was sequenced [20]. Since Arabidopsis is relatively easy to mutagenize and genetically transform, thousands of mutants and genetically modified lines are available at seed repositories. However, storage of Arabidopsis seeds is problematic because, even in optimal conditions, they deteriorate relatively quickly, losing their germination ability. This is a serious problem to seed stock centers, but also to individual researchers in order to conserve their precious Arabidopsis lines.

A series of methods collectively known as priming have been developed in order to induce a faster seed germination, a higher seed germination rate or a better seedling growth. Priming can be used to promote germination of seeds after a long storage period [21]. The positive effects of priming treatments on seed performance have been demonstrated in many species. One of the problems of priming is that some of these treatments are expensive and/or time-consuming. On the contrary, ultrasound treatments are easy, cheap (basically only the price of the ultrasound generator), and quick. Ultrasound treatments have shown promising results on other species [19], but no information is available on the effects of ultrasounds on the aged seeds of the model plant *A. thaliana*. In this study we showed that ultrasound treatment significantly increases Arabidopsis seed germination in artificially and naturally aged seeds.

The described possible effects of ultrasounds on plant tissues are multiple [19]: heat, inactivation of microorganisms and enzymes, acceleration of certain metabolic reactions, induced cavitation, etc. Cavitation consists in the formation of vapour cavities (bubbles) in a liquid that are the consequence of forces acting upon the liquid. The ultrasound induces in the liquid rapid changes of pressure that cause the formation of cavities where the pressure is relatively low. The collapse of the bubbles leads to a temperature increase and the differences in pressure may have mechanical consequences on the cellular and tissue structures. For example, if the bubbles collapse near the seed coat this may damage the surface, creating pores. Our results on SEM indicated that this is one of the effects of ultrasound in the Arabidopsis seeds. The seed coat acts as a physical barrier preventing the uptake of water and oxygen into the seed, both necessary for germination [22]. In consequence, an increase in seed coat porosity may produce an enhancement of water and oxygen intake, and, in consequence, in germination. Mechanical events such as vibration, cutting and brushing, can accelerate the germination process in *A. thaliana* L. seeds [23]. Therefore, a similar effect is expected for the opening of pores induced by ultrasounds on the seed coat of Arabidopsis seeds.

## Conclusions

In this study we investigated the effects of ultrasound treatment on *A. thaliana* germination percentages in order to determine its possible application to promote germination, in special in aged seeds. No significant positive effects were observed in recently collected seeds, but a significant increase was observed in aged seeds. The ultrasound treated seeds present a greater number of pores in the seed coat than the control ones, perhaps being this the reason of the increase in the germination rate.

## Methods

### Biological materials

*Arabidopsis thaliana* plants (Col-0 ecotype) were grown in soil in controlled environmental chambers at 20 °C with a 16/8-h light–dark photoperiod. The harvested seeds were stored at room temperature in 1.5 ml tubes.

### Ultrasound

Ultrasounds were generated with 45 kHz frequency and 0.028 W m^−3^ volumetric power in a USC-1400 ultrasonic bath (Unique, Brazil). Seed sonication was performed after cold imbibition for 4 days. Ultrasound treatments were performed in a water bath at constant temperature (24 °C) and different time periods (from 30 s to 64 min). Seeds were treated in 1.5 ml plastic tubes containing 50 µl of distillate water.

### Germination test

Germination tests were performed basically as previously reported [17, 18] using 10 replicates of 30 seeds. Seeds were placed into rolled paper towels moistened with water at a proportion of 2.5-times the dry weight of the paper towels and placed at 24 °C with a photoperiod of 16 h light per day. Final germination rates were scored by counting seeds with radicle protrusion after 7 days.

### Accelerated ageing

Artificial ageing of Arabidopsis seeds were performed according to previous reports [17, 18]. Basically, one layer of seeds were placed on a metal mesh into plastic boxes containing distilled water. These boxes were closed and placed in a chamber at 40 °C. Hence, within the boxes, the seeds were exposed to 100% relative humidity. The duration of the treatment depends on each experiment from 6 h to 4 days.

### Data analysis

The statistical analyses were done using the *t* test for 2 Independent Means. Significance level were tested at p < 0.05.

### Scanning electron microscopy

Arabidopsis seeds were dehydrated in an acetone series, critical point dried using carbon dioxide and mounted directly on stubs using double-side adhesive tape. Observations were made in a EVO MA-10 SEM.

## Abbreviation

SEM: scanning electron microscopy
